# Effects of Mindfulness Meditation on Musical Aesthetic Emotion Processing

**DOI:** 10.3389/fpsyg.2021.648062

**Published:** 2021-07-21

**Authors:** Xiaolin Liu, Yong Liu, Huijuan Shi, Maoping Zheng

**Affiliations:** ^1^Key Laboratory of Cognition and Personality (Ministry of Education), Southwest University, Chongqing, China; ^2^School of Psychology, Southwest University, Chongqing, China; ^3^Research Institute of Aesthetics Psychology of Chinese Classical Music and Basic Theory of Music Performance, Chongqing Institute of Foreign Studies, Chongqing, China; ^4^School of Music, Southwest University, Chongqing, China

**Keywords:** meditation, mindfulness, music aesthetics, music emotion, beauty

## Abstract

Mindfulness meditation is a form of self-regulatory training for the mind and the body. The relationship between mindfulness meditation and musical aesthetic emotion processing (MAEP) remains unclear. This study aimed to explore the effect of temporary mindfulness meditation on MAEP while listening to Chinese classical folk instrumental musical works. A 2 [(groups: mindfulness meditation group (MMG); control group (CG)] × 3 (music emotions: calm music, happy music, and sad music) mixed experimental design and a convenience sample of university students were used to verify our hypotheses, which were based on the premise that temporary mindfulness meditation may affect MAEP (MMG vs. CG). Sixty-seven non-musically trained participants (65.7% female, age range: 18–22 years) were randomly assigned to two groups (MMG or CG). Participants in MMG were given a single 10-min recorded mindfulness meditation training before and when listening to music. The instruments for psychological measurement comprised of the Five Facet Mindfulness Questionnaire (FFMQ) and the Positive and Negative Affect Schedule (PANAS). Self-report results showed no significant between-group differences for PANAS and for the scores of four subscales of the FFMQ (*p* > 0.05 throughout), except for the non-judging of inner experience subscale. Results showed that temporary mindfulness meditation training decreased the negative emotional experiences of happy and sad music and the positive emotional experiences of calm music during recognition and experience and promoted beautiful musical experiences in individuals with no musical training. Maintaining a state of mindfulness while listening to music enhanced body awareness and led to experiencing a faster passage of musical time. In addition, it was found that Chinese classical folk instrumental musical works effectively induced aesthetic emotion and produced multidimensional aesthetic experiences among non-musically trained adults. This study provides new insights into the relationship between mindfulness and music emotion.

## Introduction

Music, as a temporal auditory art, tends to evoke aesthetic emotions (Scherer, 2004; Frijda and Sundararaja, 2007). The aesthetic values of music are capable of inducing a much more nuanced range of emotive states than discrete emotions outlined in the traditional emotion models (Zentner et al., 2008). Previous studies suggest that music evokes emotions that are significantly different from utilitarian emotions; emotions evoked by music are more often aesthetic than utilitarian (Stecker, 2006; Nieminen et al., 2012; Silvia et al., 2015; Reybrouck et al., 2018; Menninghaus et al., 2019). Many aesthetic emotions, although positive overall, encompass negative or mixed emotional ingredients, which are measured by domain-specific models (Barrett et al., 2010; Larsen and Stastny, 2011; Swaminathan and Schellenberg, 2015; Menninghaus et al., 2019; Cowen et al., 2020). Theoretical approaches have verified that domain-specific models might be more appropriate (Zentner et al., 2008; Zentner, 2010; Zentner and Eerola, 2010) compared to the classical theories of basic emotions that concern fear, anger, or joy, for example (Ekman, 1992), or dimensional models that describe all the affective experiences in terms of valence and arousal (Russell, 2003). This study is based on the aesthetic emotion theory, which is a domain-specific model of emotion (Zentner et al., 2008; Menninghaus et al., 2019).

Previous studies have shown that the processing of music aesthetic emotion involves the multidimensional measurement of both the psychological and the behavioral phenomena (Brattico et al., 2016; Menninghaus et al., 2019). Neuroscientific evidence shows that music evokes complex emotions beyond pleasant/unpleasant or happy/sad dichotomies (Nieminen et al., 2011; Brattico et al., 2016; Menninghaus et al., 2019). The processing of music aesthetic emotion is closely related to the emotional response, aesthetic judgment, and aesthetic preference of an individual while listening to music (Schubert, 2007; Barrett et al., 2010; Nieminen et al., 2012; Brattico et al., 2013; Lee et al., 2013; Orozco, 2015). The emotional response to musical aesthetics involves two processes, emotional recognition (emotion expressed by music) and emotion experience (emotion experienced by subjects) (Schubert, 2013; Schindler et al., 2017; Schubert and North, 2020), both of which are regarded as a continuum of aesthetic emotion processing (Eerola and Vuoskoski, 2011; Swaminathan and Schellenberg, 2015; Scherer et al., 2019). A forced choice task (FCT), wherein the judges were asked to select from several pre-selected emotion labels, was used to evaluate the recognition and experience of music emotion (Laukka and Juslin, 2007; Eerola and Vuoskoski, 2011; Laukka et al., 2013). Even though the forced-choice methodology produces an ecologically valid task, the participants were further required to rate the intensity of each stimulus on the Likert scale (Laukka and Juslin, 2007). The forced-choice response paradigm is a simple, clear, and methodologically strong technique that provides robust results (Frank and Stennett, 2001, p. 1). The Geneva Emotional Musical Scale (GEMS) is an effective psychometric scale based on the theory of aesthetic emotion and employs suitable ecological musical works as stimulus materials (Zentner et al., 2008; Vuoskoski and Eerola, 2011; Trost et al., 2012). The GEMS acts as an effective tool for exploring the behavioral characteristics and psychological mechanisms of Musical aesthetic emotion processing (MAEP). It is a nine factorial music emotion model comprising wonder, transcendence, tenderness, nostalgia, peacefulness, power, joyfulness, tension, and sadness (Zentner et al., 2008; Zentner, 2010; Trost et al., 2012). Tension is an important indicator of the physiological changes in an emotional experience and reflects the degree to which the psychophysiological state of an individual could change, from relaxation to tension (Menninghaus et al., 2019; Scherer et al., 2019). Activation/arousal can be used to measure the psychophysiological and neurophysiological intensity induced by music (Barrett et al., 2010; Trost et al., 2012; Vuoskoski and Eerola, 2012; Ellison et al., 2015) and the intensity of the psychophysiological and neurophysiological activation in the domain-general model of emotion (Hölzel et al., 2011a; Bueno et al., 2015; Andreu et al., 2019; Bailey et al., 2019). To facilitate the measurement of the degree of relaxation induced by mindfulness meditation training (Dvorak and Hernandez-Ruiz, 2019; Hernandez-Ruiz and Dvorak, 2020; Loo et al., 2020) and music listening (Baylan et al., 2018; Sorensen et al., 2018), this study selected tension as an indicator of the psychophysiological response of the participants.

The evaluation of aesthetic judgment and aesthetic preference also have an important influence on the processing of the aesthetic emotion while listening to music (Brattico et al., 2016; Menninghaus et al., 2019). Beauty is the core of music aesthetics (Nieminen et al., 2012; Brattico and Pearce, 2013), which appears to be “in the interaction between the stimulus and the beholder's cognitive and affective processes” (Reber et al., 2004, p. 3). Aesthetic judgment is an important measure in experiencing the beauty of music, to better account for typical aesthetic emotions, such as admiration and awe (Juslin, 2013; Schindler et al., 2017). Liking is a comprehensive index reflecting musical preference and personality traits. Some studies have shown that liking was also significantly related to the quality of music and the listening environment (Schellenberg et al., 2008; Brattico et al., 2013). Studies have shown that music preference can effectively predict aesthetic judgments and affect the emotional arousal and intensity of musical enjoyment (Nieminen et al., 2012; Brattico et al., 2016; Menninghaus et al., 2019).

Usually, phenomena such as tension, beauty, and liking in relation to aesthetic emotion recognition and experience would be measured by means of the Likert scale (Schellenberg et al., 2008; Munar et al., 2011; Trost et al., 2012; Juslin et al., 2013; Thammasan et al., 2017; Madsen et al., 2019). Evidence shows that the conceptualization, classification, and measurement of aesthetic emotions (Zentner et al., 2008; Schindler et al., 2017; Menninghaus et al., 2019) have provided important theoretical models and measurement tools for the music aesthetic domain (Zentner et al., 2008; Brattico et al., 2009; Barrett et al., 2010; Zentner, 2010; Trost et al., 2012; Diaz, 2013; Menninghaus et al., 2019), which have had a profound impact on the study of music aesthetics and emotional processing (Nieminen et al., 2012; Brattico et al., 2016; Madsen et al., 2019).

Retrospective evaluation has generally been used to measure the behavioral characteristics of the participants in the experimental research into MAEP (Zentner et al., 2008; Trost et al., 2012; Vuoskoski and Eerola, 2012, 2017; Silvia et al., 2015). However, most of the previous empirical studies have focused on exploring causality or correlation between musical noumena and MAEP, paying little attention to the influence of mindfulness meditation on MAEP (Baylan et al., 2018; Scherer et al., 2019; Hernandez-Ruiz and Dvorak, 2020; Loo et al., 2020; Misba et al., 2020).

Mindfulness, combined with Buddhist meditation, has a long history. Mindfulness is usually defined as “paying attention in a particular way: on purpose, in the present moment, and non-judgmentally” (Diaz, 2013; Lutz et al., 2014; Rodríguez-Carvajal and Lecuona, 2014). Mindfulness meditation, as a state, trait, or clinical intervention, has been widely used in emotion regulation, attention, psychotherapy, and clinical medicine, among other fields (Brown and Ryan, 2003; Sahdra et al., 2011; Singh et al., 2014; Bueno et al., 2015; Zanesco et al., 2016; Zanesco, 2017; Andreu et al., 2019; Bailey et al., 2019). It has brought many positive effects to people, such as regulated negative emotions, enhanced self-awareness, improved well-being, enhanced psychological function, and reduced stress symptoms, and improved cognitive recovery (Brown and Ryan, 2003; Rodríguez-Carvajal and Lecuona, 2014; Tomaselli, 2014; Anderson, 2016; Bell et al., 2016; Baylan et al., 2018; Sorensen et al., 2018; Loo et al., 2020; Misba et al., 2020). Previous studies have shown a certain relationship between mindfulness meditation and specific music activities (Rodríguez-Carvajal and Lecuona, 2014). Mindfulness-based music listening can increase listening sensitivity and enjoyment (Anderson, 2016; Baylan et al., 2018), improve well-being (Brown and Ryan, 2003; Rodríguez-Carvajal and Lecuona, 2014; Sorensen et al., 2018; Loo et al., 2020), enhance body awareness and listening experiences (Diaz, 2013; Rodríguez-Carvajal and Lecuona, 2014), and decrease psychological stress and anxiety symptoms (Tomaselli, 2014). Specific musical activities, such as listening to mindfulness music, chorus training, music performance, and music creation, could induce a state of mindfulness meditation, which moderates the absorption of musical stimuli by the participants and affects their emotional experiences (Bell et al., 2016; Lynch and Wilson, 2017; Dvorak and Hernandez-Ruiz, 2019).

Previous studies have shown that mindfulness meditation could improve emotion processing (Tan et al., 2014; Bueno et al., 2015; Lei et al., 2016). Mindfulness meditation comprises a process of enhanced self-regulation that can be differentiated into distinct but interrelated components, namely, attention regulation, body awareness, emotion regulation, and a change in self-perception (Hölzel et al., 2011b). Mindfulness-based intervention contributes to inducing a positive emotional state (Brown and Ryan, 2003) and reducing negative emotions (Baylan et al., 2018). Neurophysiological evidence shows that mindfulness meditation causes structural changes in the brain regions involved in learning and memory processes, emotion regulation, and self-referential processing (Davidson et al., 2003; Hölzel et al., 2011a; Lutz et al., 2014; Davidson and Kaszniak, 2015; Tang et al., 2015). Mindfulness meditation training can be divided into three levels according to the training time (Davidson, 2010; Davidson and Kaszniak, 2015; Sayers et al., 2015; Lei et al., 2016): temporary mindfulness meditation training (3 min to 1 h), short-term mindfulness meditation training (4 days to 4 months), and long-term mindfulness meditation training (over 10 years). Studies have shown that both short-term mindfulness meditation training and long-term meditation training affect emotional processing by increasing individual mindfulness and self-awareness and enhancing individual emotional self-acceptance (Lei et al., 2016). Erisman and Roemer (2010) examined the effect of a temporary mindfulness meditation intervention on emotional response in terms of three emotions (sad, positive, and mixed), following the viewing of film clips. The results showed that temporary mindfulness meditation intervention effectively enhanced positive experiences and reduced negative experiences (Erisman and Roemer, 2010). A neuroimaging study showed that temporary mindfulness meditation training enhanced the activation of the prefrontal lobe and reduced the activation of the amygdala and the hippocampus (Lutz et al., 2014). These studies indicate that temporary mindfulness meditation training is an effective method of mood regulation and affected emotional processing (Erisman and Roemer, 2010; Lalot et al., 2014; Lutz et al., 2014; Garland et al., 2015; Lei et al., 2016).

Mindfulness meditation is the intentional awareness of internal and external happenings in the present moment, without judgment, rejection, or attachment to the moment (Kabat-Zinn, 2012; Dvorak and Hernandez-Ruiz, 2019; Hernandez-Ruiz and Dvorak, 2020). It includes two components, namely, regulating the attention of an individual to maintain the immediate experience and approaching the experiences of an individual with curiosity, openness, and acceptance (Lei et al., 2016). The non-judgment and openness experience induced by state mindfulness meditation are highly related to body awareness (Brown and Ryan, 2003; Diaz, 2013; Rodríguez-Carvajal and Lecuona, 2014; Clark et al., 2015; Anderson, 2016; Loo et al., 2020; Misba et al., 2020). Temporary mindfulness meditation training, as a relaxed state of enhanced self-regulation, effectively induces the openness of individuals to experience and non-judging of inner experience by individuals, thereby improving emotion processing (Bishop, 2004; Tan et al., 2014; Bueno et al., 2015; Lei et al., 2016). In addition, changes in time perception may be related to body awareness and openness of the internal experience induced by mindfulness meditation training, which enhances attentional function (Brown and Ryan, 2003; Kramer et al., 2013; Bailey et al., 2019). Mindfulness meditation focuses more strongly on sensory experiences and greater awareness of feelings and of body states, which leads to a slowing down of time in the present moment (Wittmann and Schmidt, 2014). Kramer et al. (2013) found that participants engaging in mindfulness training overestimated the length of time when images were visually presented to them. The authors attribute this to the temporary changes in attention and perception, wherein the participants were more aware and focused at the moment on the task at hand (Kramer et al., 2013). Overall, the maintenance of a relaxed body state and listening sensitivity induced by mindfulness meditation may have important academic and social significance for exploring temporary mindfulness meditation training and MAEP.

Although these studies show the influence of the mindfulness training level on emotional processing (Sayers et al., 2015; Lei et al., 2016), little is known about the influence of mindfulness meditation on MAEP among non-musically trained adults (Diaz, 2013), especially following exposure to the musical aesthetics of Chinese classical folk instrumental music. Thus, far, no study has explored the relationships between mindfulness meditation using natural Chinese classical folk instrumental musical works and MAEP. Past research has shown that music-specific models, emotion validation using multiple psychometric approaches, and ecologically valid music will become more common in future music emotion research (Zentner et al., 2008; Kumar and Garg, 2010; Eerola and Vuoskoski, 2013; Menninghaus et al., 2019). Accordingly, this study aimed to explore the effect of temporary mindfulness meditation training on MAEP. Music aesthetic tasks include five items, namely, recognition, experience, tension, beauty, and liking. FCT was used to evaluate between-group differences in the recognition and experience of the musical aesthetic emotion. The Likert scale is used to assess between-group differences in tension, beauty, and liking of music aesthetic tasks. After participants had listened to the music, GEMS was used to explore within-subject consistency in evaluating the music aesthetic emotions, verifying that the music emotion belongs to the category of aesthetic emotion (Laukka and Juslin, 2007; Munar et al., 2011; Brattico et al., 2013; Laukka et al., 2013). The differences in musical aesthetic tasks between the MMG and the CG will be investigated to illustrate that temporary mindfulness meditation training has an impact on MAEP, body awareness, and time perception of non-musically trained participants. Based on previous studies, we hypothesized the following:

- First, the scores of recognition, experience, and beauty in the MMG will be higher than that in the CG, while the scores of tension and liking will be lower in MAEP;
- Second, the scores of non-judging of inner experience and time perception of music in the MMG will be higher than that in the CG, while body awareness will be lower;
- Third, Chinese classical folk instrumental musical works will express and induce multidimensional aesthetic emotion.

## Methods

### Participants

Participants (*n* = 67; 45 females) without musical training were recruited through campus advertisements. They were required to abstain from taking substances or medications that could potentially influence their concentration. All participants reported that they received no training related to mindfulness meditation. In addition, they were required to disclose any history of major psychological disorders. All participants reported being right-handed, having normal hearing and speech, and a normal or a corrected-to-normal vision. This study was approved by the Southwest University Ethics Committee.

### Stimuli

#### Mindfulness Meditation Audio

The Chinese version of the mindfulness meditation script used in this study was derived from the English version of a mindfulness script (Diaz, 2010). The translation of the text was proofread and revised by two graduate students majoring in English. The audio was recorded by professionals who have been trained in meditation and yoga for 10 years, in a soundproof room using special recording software (Xunjie audiorecorder, Shanghai Information Technology Ltd., www.xunjieshipin.com/download-audiorecorder). The duration of the audio was 10 min. It was recorded in MP3 format (Maattanen, 2003).

#### Musical Stimuli

The stimuli set consisted of 15 music clips (duration 1 min) from the Chinese classical folk instrumental musical works which were taken from commercially available “kugou” music software (Version 9.1.32MAC, Guangzhou Kugou Computer Technology Co., Ltd, www.kugou.com), which is a professional online music player application. These high-quality music clips included three emotion levels (calm, happiness, and sadness) with five excerpts for each level, categorized according to the emotional valence of music (Nyklíček et al., 1997; Thayer and Faith, 2001; Trost et al., 2012; Russo et al., 2013). The emotional valence of these music materials was assessed by 50 musicians using the nine-dimensional GEMS model (Zentner et al., 2008; Zentner, 2010). In this study, calm, happy, and sad music had Cronbach's alpha values of 0.88, 0.85, and 0.87, respectively. Participants reported that all musical stimuli were unfamiliar.

### Music Aesthetics Task and Measures

Based on previous studies, we modified the task to explore the effect of mindfulness on music aesthetics. Music aesthetic tasks include five items: recognition, experience, tension, beauty, and liking. Recognition was defined as emotions expressed by the music, while experience was defined as feelings felt by oneself (Gabrielsson, 2001; Eerola and Vuoskoski, 2011; Schubert, 2013). FCT was used to evaluate the between-group differences in the recognition and experience of music aesthetic emotion. Nine types of music aesthetic emotions were chosen from the GEMS (Zentner et al., 2008) to judge recognition and experience of multidimensional music aesthetic emotions using forced choice. GEMS is only used for multidimensional measurement of music aesthetic emotion, not for measuring emotional intensity. Based on subjective judgment of the participants, the evaluation dimension of recognition and experience could consist of multiple choices. All participants were instructed to rate the scores for tension (1 = *extremely relaxed*, 9 = *extremely tense*) and beauty (1 = *extremely ugly*, 9 = *extremely beautiful*); liking was rated on a scale of 1 (*extremely*) to 6 (*not at all*) in accordance with the preference of the participants. In order to ensure the ecology and integrity of music listening as much as possible, post-evaluation was conducted to assess the differences in MAEP.

**Figure 1:**
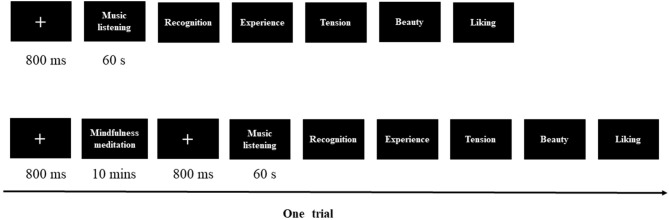
An example of a music aesthetic task.

To emphasize on the ecological validity of music listening, the temperature of the laboratory was controlled at 25°C, and the listening experiments were conducted individually for each participant in a soundproof room. E-Prime 3.0 was used to program the experiment and collect behavioral data. Fifteen music clips were presented in a pseudo-random manner to all participants, who listened to the excerpts through studio-quality headphones and were able to adjust the sound volume according to their own preferences. In the task (Figure 1), a fixation appeared for 800 ms after which the participants in the MMG were asked to practice mindfulness meditation using the audio material of temporary mindfulness meditation training, which took 10 min. At the end of the mindfulness meditation training, the participants in the MMG were presented with the following text on the screen: “Please keep the state of the mindfulness meditation as far as possible and listen to 15 music clips.” The participants in both the MMG and the CG completed the same evaluation tasks (Figure 1). The experimental instructions were as follows: “There is no experimental task while listening to the music. After listening, please answer the 5-item questions according to the task requirements.” During the task, participants were instructed to press the corresponding number button to report their evaluation results. The task consisted of 15 pieces of music and lasted about 20 min. After listening to the music, participants were requested to rate the scores of body awareness (1 = *very relaxed*, 9 = *extremely tense*) (Lutz et al., 2014; Wittmann and Schmidt, 2014) and time perception (1 = *extremely slow*, 9 = *extremely fast*) (Kramer et al., 2013; Wittmann and Schmidt, 2014). The experimental instructions for body awareness and time perception were as follows: “What is the degree of body control experienced while listening to the music?” and “What is your evaluation of the passage of musical time while listening to the music?”

### Procedure

Participants were informed that the study was about music aesthetics and that they would perform tasks on a computer. After providing written consent, participants confirmed whether they had previously received training related to mindfulness meditation before filling out the PANAS (used to assess the current mood state of the participants). Participants then performed the music aesthetic tasks based on the requirements of the mindfulness meditation state, which paid attention to the present moment and emphasized the attainment of consciousness that is non-judgmental/accepting. Finally, participants performed the post-measurement of body awareness and time perception and answered the FFMQ (used to measure the mindfulness meditation state of the participants after all music stimuli were played).

### Self-Report Measures

#### Five Facet Mindfulness Questionnaire

The Chinese version of the FFMQ, which was revised by Deng et al. (2011), has 39 items, covering five factors, namely, Observing (OB, e.g., “When walking, I will pay attention to the feeling of body parts on the move”), Describing (DS, e.g., “I am good at describing my emotions in words”), Acting with Awareness (AWA, e.g., “When I'm doing things, I'm often and easily distracted”), Non-judging of Inner Experience (NJ, e.g., “I blame myself for having irrational or inappropriate emotions”), and Non-reactive to Inner Experience (NR, e.g., “I feel my emotions and feelings, but I don't have to react to them”). The scale used a 5-point score from 1 (*not at all*) to 5 (*completely consistent*). The higher the scores, the higher the mindfulness levels. In this study, Cronbach's alpha for the FFMQ was 0.80.

#### Positive and Negative Affect Schedule

The PANAS (Watson and Clark, 1988) is a 20-item questionnaire that assesses the current mood state of the participants in terms of positive and negative effects. Participants rated the extent to which each of the 20 adjectives described their current feeling, on a 5-point scale ranging from 1 (*very slightly or not at all*) to 5 (*extremely*). Scores of the scale were totaled separately for the positive and the negative effects. As originally reported, Cronbach's alphas of positive affect ranged from 0.86 to 0.9, and Cronbach's alphas of negative affect ranged from 0.84 to 0.87 (Watson and Clark, 1988). In this study, positive affect had a Cronbach's alpha of 0.78, and the negative affect had a Cronbach's alpha of 0.73.

### Study Design and Data Analysis

In the present experiment, a 2 × 3 mixed experimental design (2 groups: MMG and CG; 3 music emotions: calm music, happy music, and sad music) was used to verify our hypotheses. The between-subject design was used for the groups and the within-subject design was used for music emotion, which had three levels of music aesthetic emotion: calm, happy, and sad. All participants were randomly assigned to MMG or CG.

**Figure 2:**
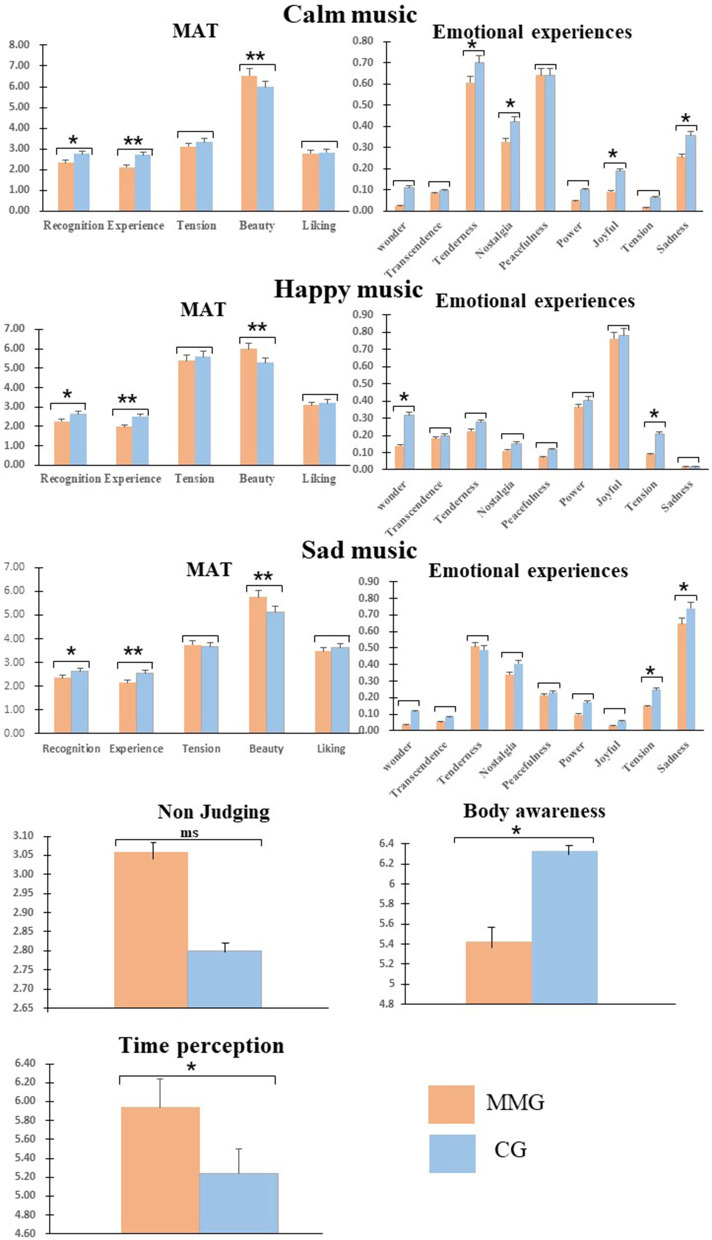
Differences between MMG and CG in the musical aesthetic tasks of music with three emotional levels, non-judging, body awareness, and time perception. MMG, mindfulness meditation group; CG, control group; MAT, Music Aesthetics Task; ms, significant marginally difference; *p < 0.05, **p < 0.01.

Independent-samples *t*-tests were used to explore the between-group differences in age, sex, PANAS, FFMQ, body awareness, and time perception (Table 1). In addition, a 2 × 3 repeated-measures ANOVA was conducted to examine the between-group discrepancies and the main effect in the five-item tasks of MAEP, with group as a between-subject factor and emotion levels as a within-subject factor (Table 1 and Figure 2). All the analyses were conducted using SPSS 22.0. The *p-*values were adjusted for sphericity using the Greenhouse–Geisser method. *Post-hoc t*-tests used Bonferroni adjustments for multiple comparisons.

**Table 1:** Demographic information and self-report results of the participants.

**Variable**		**MMG (M ± SD)**	**CG (M ± SD)**	***t***
		***n* = 33**	***n* = 34**	
Age		20.3 (1.26)	19.9 (1.23)	
Sex		Male = 10, female = 23	Male = 12, female = 21	
PANAS		PA: 2.66 (0.53); NA: 1.66 (0.47)	PA: 2.63(0.50); NA: 1.65 (0.37)	PA: 0.22; NA: 0.13
FFMQ	Sum	119.00 (8.66)	119.08 (11.84)	0.04
	OB	3.07 (0.54)	3.14 (0.58)	0.49
	DS	3.00 (0.57)	3.06 (0.58)	0.42
	AWA	3.28 (0.63)	3.40 (0.59)	0.83
	NJ^ms^	3.06 (0.43)	2.80 (0.64)	1.91
	NR	2.83 (0.36)	2.84 (0.52)	0.16
GEMS^**^		2.20 (0.51)	2.63 (0.68)	2.96
Body control^*^		5.42 (1.25)	6.33 (1.55)	2.61
Time perception^*^		5.94 (1.30)	5.24 (1.37)	2.16
**Emotion levels**		**CM**	**HM**	**SM**	**CM**	**HM**	**SM**	**CM**	**HM**	**SM**
MAT	Recognition^*^	2.33 (0.59)	2.27 (0.65)	2.36 (0.69)	2.76 (0.72)	2.64 (0.84)	2.63 (0.68)	2.68	2.00	1.62
	Experience^**^	2.11 (0.57)	1.98 (0.63)	2.14 (0.59)	2.71 (0.67)	2.51 (0.89)	2.56 (0.86)	3.89	2.82	2.30
	Tension	3.11 (1.12)	5.39 (1.31)	3.75 (1.20)	3.32 (1.20)	5.58 (1.00)	3.67 (1.20)	0.73	0.67	0.26
	Beauty^**^	6.08 (1.25)	5.45 (1.21)	5.34 (1.26)	6.34 (1.20)	5.36 (1.28)	5.25 (1.55)	0.86	0.30	0.27
	Liking	2.78 (0.56)	3.09 (0.56)	3.47 (0.64)	2.84 (0.54)	3.21 (0.75)	3.61 (0.76)	0.47	0.77	0.77

*MMG, mindfulness meditation group; CG, control group; M, mean; SD, standard deviation; PANAS, Positive and Negative Affect Scale (PA, Positive affect; NA, Negative affect); FFMQ, Five-Facet Mindfulness Questionnaire (OB, Observing; DS, Describing; AWA, Acting with Awareness; NJ, Non Judging of inner experience; NR, Non-Reactive to inner experience); GEMS, Geneva Emotional Music Scale; MAT, Music Aesthetics Task; CM, calm music; HM, happy music; SM, sad music; ms, significant marginally difference;*

* *p < 0.05,*

** *p < 0.01*.

## Results

### Self-Reported Results

The demographic information and self-report results of the participants are shown in Table 1 and Figure 2. An independent samples *t*-test found as follows: (1) The results of PANAS showed that there was no significant difference in the scores between the two groups (*p* > 0.05); (2) the post-test scores of body awareness showed that the scores of the MMG were lower than those of the CG (*t* = 2.72, *p* = 0.008, *d* = 0.67); (3) the post-test scores of musical time perception showed that the MMG experienced faster passage of time than the CG (*t* = 2.16, *p* = 0.035, *d* = 0.54); and (4) in the NJ scores, there was a significant but marginal difference between the two groups in the results of FFMQ (*t* = 1.91, *p* = 0.06, *d* = 0.47), but there was no significant difference in the scores of the other four items (OB, DS, AWA, and NR, *p* > 0.05).

### Music Aesthetic Task Results

The behavioral results of MAEP of the participants at the three emotional levels are shown in Figure 2.

#### Emotion Recognition

A repeated-measures ANOVA on emotion recognition showed the main effect of group, *F*_(1, 65)_ = 5.3, *p* = 0.02, η^2^ = 0.08. The *post-hoc t-*test showed that emotion recognition of all emotions when listening to music was lower among participants in the MMG than those in the CG. There was no main effect of music emotion levels and the interaction of group and music emotion levels (all *p* > 0.05).

#### Emotion Experience

A repeated-measures ANOVA on the emotion experience showed the main effect of group, *F*_(1, 65)_ = 10.04, *p* = 0.002, η^2^ = 0.13. The *post-hoc t*-test showed that the emotional experiences of the participants in the MMG were lower than that in the CG when listening to music, in all emotions. Results also showed the main effect of the music emotion levels, *F*_(2, 64)_ = 4.64, *p* = 0.01, η^2^ = 0.07. The *post-hoc t*-test showed that the emotional experiences while listening to calm music were significantly more than that during happy music, *p* = 0.01. The emotional experiences while listening to sad music were marginally more than that during happy music, *p* = 0.07. There was no difference between calm and sad music. There was no interaction of group and music emotion levels (all *p* > 0.05).

#### Relationship Emotion Recognition and Emotion Experience

Pearson correlation analysis showed that emotion recognition was related to emotion experience (sad music, *r* = 0.64, *p* < 0.001; calm music, *r* = 0.77, *p* < 0.001; happy music, *r* = 0.76, *p* < 0.001).

#### Tension

A repeated-measures ANOVA on tension showed the main effect of music emotion levels, *F*_(2, 64)_ = 102.53, *p* < 0.001, η^2^ = 0.61, with ratings of tension in happy music > sad music > calm music. The score of happy music was significantly greater than that of sad music, and the score of sad music was significantly greater than that of calm music (all *p* > 0.001). There was no main effect of group and the interaction of group and music emotion levels (all *p* > 0.05).

#### Beauty

A repeated-measures ANOVA on beauty showed a main effect of the group, *F*_(1, 65)_ = 8.35, *p* = 0.005, η^2^ = 0.11. The *post-hoc t-*test showed that participants rated the beauty of music significantly higher in the MMG than in the CG for all three music emotions (all *p* < 0.05). Results also showed the main effect of music emotion levels, *F*_(2, 64)_ = 18.92, *p* < 0.001, η^2^ = 0.37, with ratings of beauty in calm music > happy music and sad music. Meanwhile, there was no difference between happy music and sad music (all *p* < 0.05). There was no main interaction of group and music emotion levels (all *p* < 0.05).

#### Liking

A repeated-measures ANOVA on liking showed the main effect of music emotion levels, *F*_(2, 64)_ = 37.61, *p* < 0.001, η^2^ = 0.37, with ratings of liking in calm music > happy music > sad music. There was no main effect of group and interaction of group and music emotion levels (all *p* > 0.05).

## Discussion

In our novel examination of the influence of temporary mindfulness meditation training on MAEP, the hypotheses were partially confirmed. In line with our expectations, significant differences in recognition, experience, and beauty between the two groups were influenced by temporary mindfulness meditation training. Temporary mindfulness meditation decreased the negative emotions (e.g., tension, sadness in sad music) of happy and sad music and the positive emotions (e.g., wonder, tenderness, and joy) of calm music during recognition and experience and improved the experience of beauty in MAEP. The post-test results showed that maintaining a state of mindfulness enhanced body awareness and led to experiencing a faster passage of musical time while listening to music. Although there were no statistically significant between-group differences in tension and liking, the scores of all aesthetic dimensions in the MMG were lower than those in the CG, which suggested that temporary mindfulness meditation training might affect music aesthetic emotion. Additionally, the GEMS findings showed that Chinese classical folk instrumental musical works induced aesthetic emotions and produced multidimensional emotional experiences.

The behavioral results showed that the rating scores of emotion recognition, emotion experience, and evaluation of beauty and liking effectively measured the differences in the processing of music aesthetics in individuals with no musical training. Previous studies showed that temporary mindfulness meditation affected negative and positive emotion processing (Arch and Craske, 2006; Erisman and Roemer, 2010; Lalot et al., 2014; Lei et al., 2016). Erisman and Roemer (2010) found that temporary mindfulness meditation intervention effectively enhanced positive experience and reduced negative experience using the stimuli with three emotions (sad, positive, and mixed) (Erisman and Roemer, 2010). However, a few studies also investigated the effect of mindful attention on positive emotions using positive stimuli; their results showed that participants without previous mindfulness training reported lesser positive emotions (Arch and Craske, 2006; Lalot et al., 2014). Consistent with previous studies (Arch and Craske, 2006; Erisman and Roemer, 2010; Lalot et al., 2014), the MMG showed an effectively decreased music aesthetic emotion during recognition and experience as well as a decrease in the negative emotional experiences of happy and sad music. Compared with the MMG, the CG reported more positive emotional experiences (e.g., wonder, tenderness, and joy) in the emotional experiences of calm music. This may be due to the non-judgment of the inner experiences induced by temporal mindfulness meditation (Lei et al., 2016; Hernandez-Ruiz and Dvorak, 2020); the non-judging of inner experience refers to taking a non-evaluative stance toward thoughts and feelings (Baer et al., 2008). In previous studies, mindfulness meditation, as an effective form of regulating the body and mind and promoting health, had a significantly positive effect on regulating negative emotions, attention, emotional stability, awareness, and empathy experience (Erisman and Roemer, 2010; Tan et al., 2014; Bueno et al., 2015; Clark et al., 2015; Lei et al., 2016; Ren et al., 2018; Andreu et al., 2019; Bailey et al., 2019). Temporary mindfulness meditation training influenced aesthetic emotion recognition and experience and enhanced the experiences of musical beauty in individuals with no musical training, which is consistent with a few previous research (Bueno et al., 2015; Sayers et al., 2015; Lei et al., 2016). Temporary mindfulness meditation training, as a relaxed state of listening to music, provides us with a new method of listening and helps us to experience music in depth and generate aesthetic judgments.

Our results showed that emotion recognition was related to emotion experience. When exposed to musical aesthetics, the processing of the aesthetic emotions of individuals is affected by emotional experience, aesthetic judgment, and aesthetic preferences (Schubert, 2007; Barrett et al., 2010; Nieminen et al., 2012; Brattico et al., 2013; Lee et al., 2013; Orozco, 2015). Previous studies have found more similarities than differences between emotion recognition and experience, which are seen as a continuum (Gabrielsson, 2001; Eerola and Vuoskoski, 2011; Schubert, 2013) and which have an important influence on the judgment of music aesthetics (Eerola and Vuoskoski, 2011; Swaminathan and Schellenberg, 2015; Scherer et al., 2019). Previous studies have also shown that individuals could empathize with music and produce an individualized aesthetic experience by actively participating in music or by being deeply attracted to music. Therefore, emotion experience is more important than emotion recognition in music aesthetics (Madsen et al., 1993; Brattico et al., 2009; Paul, 2009; Menninghaus et al., 2019; Schubert and North, 2020). However, in the judgment of music emotion, perception and evaluation of music emotion of individuals are affected by subjective experience, which has a direct impact on music aesthetic judgment (Schubert, 2007; Kumar and Garg, 2010; Droit-Volet et al., 2013). In music aesthetics, recognition and experience of music aesthetic emotion are both independent and mutually influential (Gabrielsson, 2001; Schubert, 2007, 2013; Schindler et al., 2017).

In addition, tension, beauty, and liking are also important measurement dimensions of music aesthetic processing (Brattico et al., 2009, 2017; Schindler et al., 2017; Menninghaus et al., 2019; Cowen et al., 2020). Previous studies have shown that tension, emotional intensity, and activation are different measurement dimensions of subjective feelings (Zentner et al., 2008; Zentner, 2010; Menninghaus et al., 2019). This study explored how individuals with no musical training experienced a change from tension to relaxation induced by calm, happiness, and sadness in a state of mindfulness meditation. Some studies also showed that mindfulness meditation can effectively promote relaxation in individuals (Bueno et al., 2015; Tang et al., 2015; Lei et al., 2016; Bailey et al., 2019). Therefore, this study chose tension as the measurement index of relaxation and emotional intensity. Although there is no significant difference in the tension between groups, the score of tension in the MMG is slightly lower than that in the CG, which indicated that a single session of mindfulness training is not enough to promote the relaxation of aesthetic emotional experience. Further study using short-term mindfulness meditation training on tension is needed. Beauty is influenced by the interaction between an individual and music, which reflects the self-evaluation of an individual of the beauty and ugliness of the musical stimulation and affects, or even determines, the liking for music aesthetics of an individual (Reber et al., 2004; Nieminen et al., 2012; Schubert and North, 2020). Our results showed that temporary mindfulness meditation training contributes to improving the aesthetic experience of the participants in MAEP, which indicates that individuals could experience more beauty by listening to music in a relaxed physical state. Liking is influenced by the music preference and personality traits of an individual (Brattico et al., 2016; Menninghaus et al., 2019), as well as by the quality of the music and the listening environment (Schellenberg et al., 2008; Brattico et al., 2013). This experiment was conducted in a quiet, soundproof room, stimulated by traditional Chinese folk music with high ecological quality, and played with high-quality audio equipment. The results showed that there was no significant difference in liking between groups, which may be due to the influence of music preference and personality traits of individuals (Brattico and Pearce, 2013; Menninghaus et al., 2019).

Previous studies showed that individuals could maintain a state of mind and body that focused on the present moment and could be open to more positive experiences while maintaining a state of inner experience without judgment (Clark et al., 2015; Sayers et al., 2015; Lei et al., 2016). Our results showed that, compared with the CG, the MMG maintained a more relaxed awareness of body control and experienced a faster passage of the musical time while listening to music. The reduction in body control in the MMG may reflect maintaining a relaxed state while listening to music (Diaz, 2013; Rodríguez-Carvajal and Lecuona, 2014). This may be related to the relaxation and positive emotional experiences induced by mindfulness meditation (Rodríguez-Carvajal and Lecuona, 2014; Hernandez-Ruiz and Dvorak, 2020). Furthermore, some studies showed that time perception is intimately linked to affective states, which might alter the sense of time (Droit-Volet et al., 2013; Kramer et al., 2013; Wittmann and Schmidt, 2014). The faster time perception of music in the MMG may be due to moment-to-moment awareness in the positive emotional experience (Diaz, 2013; Droit-Volet et al., 2013; Rodríguez-Carvajal and Lecuona, 2014).

The following evidence confirms our hypothesis: Chinese classical folk instrumental musical works, which are consistent with Western classical music (Laukka and Juslin, 2007; Paul, 2009; Larsen and Stastny, 2011; Trost et al., 2012; Brattico et al., 2013; Eerola and Vuoskoski, 2013; Juslin et al., 2013; Madsen et al., 2019), effectively induce aesthetic emotion and produce two or more aesthetic emotion experiences among non-musically trained adults. Consistent with the previous findings regarding music aesthetics, this study selected three emotional levels of calm, happiness, and sadness with Chinese classical folk instrumental musical works as stimulus materials. Participants were not familiar with the materials used in this study. Previous studies have found that listening to negative emotional music induced by anger and fear for a long time may have a negative effect on the experience of individuals and may even produce a sense of disgust and a negative effect on the music aesthetics of individuals (Zentner et al., 2008; Zentner, 2010). By contrast, sad music based on personal mood and preference can generate positive effects (Brattico et al., 2016; Schubert, 2016; Garrido, 2017; Eerola et al., 2018). This study showed the following: (1) in terms of experience, the dimension of calm music was significantly greater than happy music, the dimension of happy music was marginally smaller than that of sad music, and there was no difference between calm music and sad music; (2) in terms of tension, the effect of calm music was significantly lower than sad music and happy music, and sad music was significantly lower than happy music; (3) in the assessment of beauty, there were noteworthy discrepancies in the three emotion levels, namely, calm music was higher than happy music and sad music, while happy music was slightly higher than sad music; and (4) in ratings of liking, calm music was higher than happy music and sad music, happy music was higher than sad music, and sad music was rated as “not very liked.” According to the mood congruence theory (Lee et al., 2013), liking of sad music might be affected by the positive mood of the participants, which indicates that one-off temporary mindfulness meditation training is not sufficient to have an impact on their preference.

## Conclusion

To our knowledge, this is the first study investigating the influence of mindfulness on MAEP in individuals with no musical training. The findings contribute to existing literature by identifying the relationship between mindfulness and musically induced emotion. Importantly, this study provided new evidence of the aesthetic responses and new insights into the association between recognition and experience of aesthetic emotion based on Chinese classical folk instrumental musical works. Some limitations of this study has been discussed. First, the sample size was comparatively small for a behavioral study. Future studies should consider larger samples to further explore the MAEP in individuals who have no musical training. Second, this study was based on individuals who had no musical training. Future studies should investigate how mindfulness meditation affects the aesthetic emotion processing of musicians. Third, we relied on self-reports, which could not provide an objective index. As such, future studies should explore neural markers or brain activities using event-related potential (ERP) or functional magnetic resonance imaging (fMRI). Finally, future research needs to explore the influence of individual music preference and different personality traits on music aesthetic processing based on temporary mindfulness meditation, and, further, provide relevant behavioral and neurophysiological evidence. As some researchers expect, future research on the medium- and long-term effects of aesthetic experience on physical and mental health, well-being, and cognitive function may benefit from regarding aesthetic emotion as an important variable. Music could thus certainly offer highly effective and pleasurable tools to further promote well-being and health (Vuilleumier and Trost, 2015; Lynch and Wilson, 2017; Sorensen et al., 2018; Menninghaus et al., 2019). In the future, in-depth research on the effects of mindfulness meditation training on aesthetic emotion will be conducive to a better understanding of the functioning and cultivation of a healthy mind (Hölzel et al., 2011b; Bueno et al., 2015; Loo et al., 2020; Misba et al., 2020).

In conclusion, the findings of this study suggest, that whether during the recognition or the experience process, music emotions are multidimensional. Temporary mindfulness meditation decreases negative emotional experiences of happy and sad music and positive emotional experiences of calm music and promotes beautiful musical experiences in individuals with no musical training. Maintaining a state of mindfulness may enhance body awareness and lead to experiencing faster passage of musical time while listening to music. Mindfulness meditation, as a positive state of listening to music, provides a new avenue by which the beauty of music could be enjoyed. This is the first study exploring the effect of mindfulness on MAEP and provides new directions for the further studies of musical aesthetics.

## Data Availability Statement

The original contributions presented in the study are included in the article/Supplementary Material, further inquiries can be directed to the corresponding author/s.

## Ethics Statement

The studies involving human participants were reviewed and approved by Research Project Ethical Review Application Form, Faculty of Psychology, Southwest University. The patients/participants provided their written informed consent to participate in this study.

## Author Contributions

XL, YL, and HS conceptualized and designed this study and wrote the manuscript. MZ reviewed the manuscript. All authors have read and agreed to the published version of the manuscript.

## Conflict of Interest

The authors declare that the research was conducted in the absence of any commercial or financial relationships that could be construed as a potential conflict of interest.
